# The Heterogeneity of Post-Menopausal Disease Risk: Could the Basis for Why Only Subsets of Females Are Affected Be Due to a Reversible Epigenetic Modification System Associated with Puberty, Menstrual Cycles, Pregnancy and Lactation, and, Ultimately, Menopause?

**DOI:** 10.3390/ijms25073866

**Published:** 2024-03-30

**Authors:** David A. Hart

**Affiliations:** Department of Surgery, Faculty of Kinesiology, and McCaig Institute for Bone and Joint Health, University of Calgary, Calgary, AB T2N 4N1, Canada; hartd@ucalgary.ca

**Keywords:** menopause, post-menopausal diseases, disease risk, reproduction, species survival

## Abstract

For much of human evolution, the average lifespan was <40 years, due in part to disease, infant mortality, predators, food insecurity, and, for females, complications of childbirth. Thus, for much of evolution, many females did not reach the age of menopause (45–50 years of age) and it is mainly in the past several hundred years that the lifespan has been extended to >75 years, primarily due to public health advances, medical interventions, antibiotics, and nutrition. Therefore, the underlying biological mechanisms responsible for disease risk following menopause must have evolved during the complex processes leading to *Homo sapiens* to serve functions in the pre-menopausal state. Furthermore, as a primary function for the survival of the species is effective reproduction, it is likely that most of the advantages of having such post-menopausal risks relate to reproduction and the ability to address environmental stresses. This opinion/perspective will be discussed in the context of how such post-menopausal risks could enhance reproduction, with improved survival of offspring, and perhaps why such risks are preserved. Not all post-menopausal females exhibit risk for this set of diseases, and those who do develop such diseases do not have all of the conditions. The diseases of the post-menopausal state do not operate as a unified complex, but as independent variables, with the potential for some overlap. The how and why there would be such heterogeneity if the risk factors serve essential functions during the reproductive years is also discussed and the concept of sets of reversible epigenetic changes associated with puberty, pregnancy, and lactation is offered to explain the observations regarding the distribution of post-menopausal conditions and their potential roles in reproduction. While the involvement of an epigenetic system with a dynamic “modification-demodification-remodification” paradigm contributing to disease risk is a hypothesis at this point, validation of it could lead to a better understanding of post-menopausal disease risk in the context of reproduction with commonalities may also lead to future improved interventions to control such risk after menopause.

## 1. Purpose: Discuss the Potential Role of Variables Leading to Disease Risk in the Post-Menopausal State Having Important Roles during the Reproductive Stage of the Life Cycle, and Then Focus on Potential Mechanisms That Are Involved

Most articles regarding diseases arising in the post-menopausal phase of a female’s life cycle focus on individual diseases (i.e., osteoporosis, cardiovascular disease, obesity, dementia, osteoarthritis). They usually do not view them in an integrated manner, nor do they ask the questions “why these conditions?”, “why only a subset of females?”, or “why do individual females not have all of these conditions?”. This review article attempts to take the position that these are not just diseases, but the underlying mechanisms are advantageous to the reproduction and survival of the species down through evolution during the critical reproductive phase of the life cycle. A potential mechanism that attempts to explain commonalities in post-menopausal disease risk is also advanced. This review searched primarily PubMed (1966–17 March 2024) and Google for relevant peer-reviewed literature using >25 terms associated with menopause, post-menopausal diseases, epigenetics and menopause, epigenetics and post-menopausal diseases, reproduction and epigenetics, sex hormones and lifespan and epigenetics, endothelial cells and biological regulation, and diet and menopause and post-menopausal conditions. References included primary publications and recent reviews where appropriate.

## 2. Background/Introduction

Menopause, with the loss of menstrual cycles and systemic hormonal fluctuations, and cessation of the reproduction stage of the life cycle for females, can have profound effects on the quality of life and the increased risk for the development of diseases and conditions in subsets of females. This transition and the associated changes have been the focus of considerable attention both from the health professional community, researchers, and those focused on societal aspects of aging and healthy aging. With the extension of the lifespan to ~80 years, many females can live a significant percentage of their lives in the post-menopausal state, and, thus, complications and disease risk in the post-menopausal state can greatly impact their lives.

This was not always the case throughout evolution to *Homo sapiens*, as the average lifespan was likely much less (~40–45 years of age), although certainly some lived longer (discussed in [1,2]). This shortened lifespan through much of evolutionary history was likely influenced by infant mortality, mortality in childbirth, disease, food insecurity/nutrition, accidents, and predators. The extensions to the lifespan over the past few hundred to thousand years can likely be attributed to public health initiatives, medical interventions, antibiotics, food quality and security, improved infant survival, and decreased mortality in childbirth. Thus, for much of evolutionary history, the risk for post-menopausal diseases was either mostly non-existent or only a few individuals were affected. Furthermore, some conditions, such as dementia, often occur later in life, so even fewer individuals would be overtly affected. As the post-menopausal state is superimposed on the aging background, in the past, one would potentially be unable to segregate aging effects from the post-menopausal risk factors, and while they are integrated, epidemiologically they can be somewhat segregated.

Of note, of the several conditions or diseases associated with the post-menopausal state (i.e., osteoporosis, osteoarthritis, cardiovascular disease, obesity, dementia) [3,4,5,6,7,8,9], reviewed in [10,11], most women do not have all of the indicated conditions or the majority of them. However, there can be overlap between some of these conditions where some of the disease risks appear to be interdependent [7,12]. In addition, for conditions such as osteoporosis, there is extensive heterogeneity in the rate of bone loss and where the bone is lost, so not only are only a subset of women affected, but details regarding how they are affected vary (discussed in [1]). For some of these conditions (i.e., cardiovascular diseases, osteoarthritis) females appear to be somewhat protected from such conditions prior to menopause and then lose this “protection” following menopause [13]. In contrast, loss of bone and development of osteoporosis (OP) may result from a loss of regulation of a system that is used during reproduction to access calcium stores for fetal growth (discussed in [1]). This regulatory system regarding calcium mobilization can result in OP during pregnancy and lactation [14]; discussed in [1]. The links to obesity associated with the post-menopausal state may relate to the regulation of metabolism and the efficiency of energy storage to survive food insecurity, particularly when pregnant over winter with food insecurity is more prevalent. Dementia can be either vascular or non-vascular, so there could also be an overlap between post-menopausal risk for cardiovascular disease and some forms of dementia [9]. Prior to menopause, the incidence of osteoarthritis (OA) in men and women is ~1/1, while after menopause, the incidence of OA in women exceeds that of men (~3/1) (discussed in [1,10]). What the molecular basis is for this increase is unknown, but it could relate in part, to the development of obesity as individuals with obesity can develop metabolic OA (discussed in [10]). While there may be overlap between the various conditions associated with disease risk in the post-menopausal state, only subsets of post-menopausal females are clinically affected. That is, only ~30% of females exhibit OP [15], and in that subset, the extent or progression of the condition is quite variable (discussed in [1]). Thus, females with diagnosed OP may lose bone rapidly or slowly. Therefore, even those with OP are quite heterogenous and the progression of OP is complex, likely involving a set of genes, with some involved in the initiation of the condition and others involved in the rate of disease progression. The genes involved in the latter may not be directly related to the post-menopausal state, as astronauts, mostly males thus far, also lose bone in a heterogenous manner while in microgravity (discussed in [11,16]).

## 3. Variables and Factors Influencing Post-Menopause Diseases and Conditions

A number of variables such as early menopause [17] and reproductive parity [18,19,20] have been reported to influence some post-menopausal conditions such as OP, but not all studies are in agreement [21]. Early menopause can lead to OP and a high parity number can also lead to OP. The latter may be the result of repeated pregnancy and lactation bone loss without ample time to recover. However, the studies by Seo et al. [19] and Yang et al. [20] were performed with Asian populations while that of de Bakker et al. [21] was with a Canadian population that was likely mainly Caucasian, so there could potentially be racial/ethnic differences in this regard. Early menopause, defined as menopause onset < 40 years of age, has been reported to increase the risk of developing dementia and cognitive decline (reviewed in [22,23,24]).

Co-morbidities such as HIV [25] and rheumatoid arthritis [26] have been reported to lead to higher incidence of OP or fragility fractures, respectively. Other co-morbidities such as COPD [27], kidney disease [28], and periodontitis [29] have also been reported to influence OP. While not apparently influencing the incidence of OP but aggravating the condition, the basis may be related to the inflammation associated with such conditions, as inflammation can also affect bone (reviewed [30]). Some of these inflammatory influences may be associated with an age-related low level of chronic inflammation (inflammaging) [31].

Other factors that may also contribute to OP risk and progression include periostin levels associated with endocrine disease [32], the gut microbiota [33,34], anti-Mullerian hormone [35,36], and genetic factors [37], reviewed in [38,39], and mediators such as irisin [40]. Vascular dysfunctions such as varicose veins have also been implicated in the development of OP [41]. Therefore, there are numerous risk factors for the elaboration of OP and its progression, but the basis for why a specific subset of women (~30%) develops clinically defined OP remains unknown.

Osteoporosis after menopause onset is not the only condition that appears to be influenced by inflammatory processes. Dementia, including Alzheimer’s disease (AD), is also more common in post-menopausal women than in men (discussed in [42]). Similar to OP, AD also appears to be influenced by inflammatory processes [42,43,44]. Some of this influence may be at the level of the vascular component of the system as sex hormones contribute to cerebrovascular function [9,45]. In support of this concept, it has been reported that adiponectin, an anti-inflammatory cytokine produced mainly by adipose tissue may contribute to a positive effect on post-menopausal cognitive decline [46]. However, it remains to be determined whether inflammatory processes primarily influence disease progression rather than trigger the disease itself. Interestingly, in mouse models, vitamin D deficiency can exacerbate AD-like disease by reducing antioxidant capacity in the brain [47], again supporting a role for inflammation-like processes in disease progression but not initiation.

As some people have elevated tangles and tau protein levels but no loss of cognition, and others have cognitive decline but without elevated tau [48], there may be multiple steps in the loss of cognition and disease development, one based on some primary initiating step followed by a secondary step, potentially involving inflammation. As there also appears to be a subset of post-menopausal patients who have a vascular form of the disease, the first step(s) in disease induction may involve different components and there are multiple ways to develop the condition. However, much of mainstream research has focused on amyloid dysfunction in AD, with recent drugs designed to assist in the removal of such proteins [49], but the effects of the drugs are quite modest. Therefore, the molecular basis for the development of dementia in a subset of the post-menopausal population remains largely unknown.

Prior to menopause, women and men appear to be equally affected by OA, but after menopause, the incidence in women exceeds that in men (discussed in [1,2,10,50]). Whether the development of OA, particularly in the knee, in the post-menopausal state is a unique subset of OA patients is unknown, but the possibility has been raised [10]. However, it is clear that OA is an inflammatory disease (discussed in [51,52,53,54]) and in early post-traumatic knee injuries leading to OA in preclinical models, treatment to inhibit inflammation ameliorates the development of the condition [55,56,57]. Furthermore, metabolic osteoarthritis has an inflammatory component [58,59,60] and this may relate to post-menopausal women with obesity.

Before menopause, women are reported to be somewhat protected from cardiovascular diseases (CVD), but after menopause, there is an increased risk for such conditions (discussed in [61,62,63,64]). Some of this increase may be linked to the co-development of increases in body fat (discussed in [64]). As with some of the conditions discussed above, aspects of the CVD risk after menopause may be again linked to inflammatory processes [65]. This may be related to calcium levels [66], as calcium is a key regulator and may be a link between OP and heart disease. Interestingly, genes involved in some inflammatory pathways have been reported to be biomarkers for coronary heart disease in post-menopausal Thai women [67]. This may relate to the development of hypertension in the post-menopausal state for some women [68,69].

A subset of women with rheumatoid arthritis (RA), an autoimmune inflammatory disease, are also prone to developing cardiovascular disease [70,71,72,73]. Of interest is the fact that ~70% of female RA patients undergo a remission of their disease when pregnant and disease activity is also influenced by the menstrual cycle (reviewed in [74]). Why only ~70% of female RA patients undergo remission is unknown, but certainly, pregnancy is known to lead to a downregulation of inflammatory responses, possibly to better carry an allogeneic fetus. Whatever the mechanism(s) that are involved, such findings do indicate that the regulation of inflammatory processes is different in males and females, and inflammatory processes developing in the post-menopausal state may be indirectly related to reproductive functions [75,76]. Thus, future considerations should investigate the potential efficacy of anti-inflammatory therapies in the onset and progression of several of the post-menopausal onset conditions and diseases.

## 4. Summary of Points Potentially Relevant to Post-Menopausal Conditions

From the above discussion, a number of salient points regarding post-menopausal conditions and diseases have to be reconciled in order to understand how they arise, and their potential role after puberty and prior to menopause. These points include the following:

- Not all women experience these conditions (i.e., osteoporosis, osteoarthritis, dementia, obesity, cardiovascular disease) after menopause, and those that do usually only experience a subset of them.
- For some of the conditions, such as OP and others, there appears to be a genetic component to disease risk, as it “runs in families”.
- Most if not all of the post-menopausal conditions can be linked directly or indirectly to reproductive activities such as pregnancy and lactation.
- Early menopause and elevated parity contribute to OP risk and low bone density, respectively. Early menopause is also linked to other post-menopausal conditions [77].
- Inflammation appears to be a “catalyst” for post-menopausal disease progression but possibly not an inducer of the conditions.

Additional potentially relevant points include the following:

AA. Epigenetic modifications of the genome via direct methylation or indirectly via modification of histones, as well as modification of RNA can occur under a variety of circumstances (discussed in [78,79,80,81,82]). Many of these alterations are reversible modifications that allow for modulation and regulation of gene expression and function. Several of such alterations have been implicated in diseases or conditions relevant to menopause, such as osteoporosis and calcium signaling [83,84,85], cardiovascular disease [86,87], osteoarthritis [88,89,90], the nervous system and the brain [91,92,93,94], obesity [95,96], and vascular inflammation [97].
BB. The onset of puberty is associated with epigenetic alterations to a variety of genes (discussed in [1,2]). Epigenetic modifications also occur in the brain during development and aging [98,99].
CC. Epigenetic modifications occur with the onset of menopause [1,100] and in those with post-menopausal conditions such as OP (discussed in [1,11]), cardiovascular conditions [101], and others [98,102]. However, in many of these conditions, their relationship to disease induction versus arising as a consequence of the diseases/conditions is not known.
DD. Treatment of individuals undergoing gender transitioning with hormones such as estrogen leads to epigenetic modification of the genome of blood cells [103].
EE. Hormonal changes associated with pregnancy involve several molecules in addition to estrogen (discussed in [104,105,106,107,108]).
FF. Epigenetic modifications occurring in the white blood cells of astronauts while in space are reversible following a return to Earth [109], as discussed in [16].
GG. Young women are heterogeneous with regard to menstrual cycle-associated alterations. Approximately 20% do not exhibit alterations to knee joint laxity across the menstrual cycle in spite of similar changes in estrogen levels to the 80% that do exhibit changes [110,111].
HH. Pregnancy is associated with a downregulation of inflammation in a majority (~70%) of those with inflammatory autoimmune diseases such as rheumatoid arthritis, and disease activity is influenced by menstrual cycle variations in hormone levels [74].
II. Pregnancy is associated with cognitive changes [104].
JJ. Endothelial cells of the vasculature can differentiate to form paracrine systems with other cells in a local environment and thus form unique paracrine systems with distinctive characteristics (reviewed in [112]; discussed in [113,114]).

## 5. Involvement of Epigenetic Modification across the Lifespan of Females

Epigenetics, the modification of DNA and DNA activity, can occur across the lifespan [115,116] and can occur during aging [117], often in response to environmental stressors [118]. Such changes can be reversible [16,109,119], and thus, such a system is dynamic and not unidirectional. Some of the complexity of epigenetic modifications occurring in females across the lifespan are detailed below.

### 5.1. During Puberty and Gender Transitioning

After a period of growth and maturation, females undergo puberty at ~11–13 years of age. Puberty is accompanied by a number of epigenetic modifications in a variety of tissues in preparation for reproduction. The affected tissues can be in the brain [120], specifically the hypothalamus [121,122,123], peripheral blood leukocytes [124], adipocytes [102], and mammary glands [125], to name several. Additional genetic and epigenetic factors have been associated with precocious puberty [126,127,128]. Using saliva, Stueve et al. [126] reported that epigenetic modification of specific genes such as CYP19A1 may be a biomarker for pubertal timing. Thus, a variety of tissues are affected primarily and secondarily during puberty onset. Interestingly, using hormonal therapy for gender transitioning can also lead to epigenetic modifications of a variety of cells [103] and is being investigated in tissues [129]. Therefore, the onset of puberty leads to a variety of epigenetic modifications to both prepare for reproductive activities and also the growth and maturation that occurs during the post-puberty adolescent period.

### 5.2. During Pregnancy and Lactation

A large number of adaptations occur in a female when she becomes pregnant. Not only is she carrying a fetus that is a histoincompatible allograft (e.g., a “foreign graft”) that must be implanted, but also requires adaptations that assist in nurturing its growth and maturation, as well as accommodating stresses on a variety of the mother’s organs [130,131]. Some of these adaptations appear to be manifested by epigenetic modifications [130,131,132,133,134,135]. However, some long-term epigenetic modifications may also contribute to adverse risks of pregnancy leading to pathological responses [136,137], but others appear to be transient and reversed during the postpartum period [131]. 

Subsequently, post-birth, the induction of lactation appears to be associated with epigenetic modifications to cells in the mammary glands, leading to the generation of milk [125,138,139,140]. Evolutionarily, this of course also required the release of calcium from bones to contribute to milk formation [141,142,143]. In a subset of women, this can lead to overt OP [143].

Many of the studies reported above for humans used white blood cells to assess the epigenetic changes, so it remains to be largely determined as to whether there are unique epigenetic modifications that are tissue-specific and which ones may be transient and reversible after pregnancy and lactation, and which may be more permanent. In addition, the influence of parity on the extent and permanence of epigenetic modifications occurring during pregnancy and lactation has not been well studied.

### 5.3. During Menopause and during Post-Menopausal Interventions

Aging in females can lead to DNA methylation alterations at the level of the ovaries, impacting fertility [100,144]. The relationship of such changes to menopause onset remains undefined. However, with the advent of menopause, a variety of conditions/diseases can arise that impact a diverse set of tissues (e.g., bone, brain, heart, adipose, muscles, and tissues of the musculoskeletal system). Accompanying some of these diseases or risk for diseases are epigenetic modifications, usually detected with white blood cells in human females. For example, hypomethylation of Alu elements in the DNA has been reported for females with osteoporosis [145], DNA methylation alterations were reported for post-menopausal females associated with metabolic and immunological systems [146], and identification of an epigenetic “signature” for CVD in post-menopausal females has been reported [147]. Whether similar epigenetic modifications are also evident in specific target tissues remains largely unknown.

However, efforts to overcome the loss of sex hormones at menopause with hormone replacement therapy (HRT) have had a “checkered” history, with the pros and cons debated regarding the risk factors (discussed in [148,149,150,151]), including cancer risk [149,152,153]. Over the ensuing years, lessons have been learned regarding dosage, composition, when to initiate HRT, and for how long to mitigate some of the risks in these subsets of females, and this has led to more females taking HRT for treatment of post-menopausal conditions.

In menopausal females, HRT is reported to inhibit/prevent OP [154,155], metabolic syndromes [156], and loss of intervertebral disc height [157], as well as not posing a risk for breast cancer [158], but evidence for a role in protection at the level of OA [159], CVD [160,161] and dementia/Alzheimer’s disease [162] is still controversial via mixed results of studies over the past 25 years.

Using nucleated blood cells, a few reports have indicated that HRT use can lead to epigenetic modifications such as DNA methylation [163,164]. While interesting, these studies do not address the issue of tissue-specific epigenetic modifications.

Finally, non-drug protocols that instead use exercise protocols, which have been shown to decrease risk for a variety of post-menopausal diseases (reviewed in [1,2,11]), have been reported to lead to alterations in epigenetic modifications in blood cells of post-menopausal females with risk for diabetes [165] or in a population of older females without Alzheimer’s disease [166]. In the latter report, some specific genes became hypomethylated while others became hypermethylated, so the changes were not unidirectional.

Based on the above discussion, females undergo a series of epigenetic modifications at distinct times during their lifespan. However, the inter-relationships between those modifications (e.g., those occurring at puberty impacting those during pregnancy, those occurring during pregnancy impacting those occurring at menopause) remain to be determined. Furthermore, in human populations, much of the literature has focused on epigenetic changes associated with white blood cells, so tissue-specific effects regarding the genes and molecules affected largely remain to be elucidated.

## 6. The Epigenetic *Modification/Demodification/Remodification* Hypothesis to Address the Development of Post-Menopausal Diseases and Disease Risk

From the above discussion, females undergo a number of epigenetic modifications during their life span, with many associated with events related to sex hormones and reproductive functions (e.g., puberty, pregnancy, lactation, and menopause). Furthermore, some of these event-dependent modifications appear to be transient and reversible, such as those following pregnancy and lactation [131]. Such reversals of epigenetic modifications can also occur in other circumstances so are not unique to pregnancy.

Following menopause, subsets of women develop an increased risk of developing unique diseases or conditions that relate directly or indirectly to reproductive functioning (e.g., osteoporosis, dementia, obesity, and cardiovascular disease). Not all females are affected, and some females may have more than one condition, but evidence that a subset has all of the conditions could not be found. Nearly all of the conditions/diseases are usually treated as independent conditions, in part because of the way medicine is organized and specialties are focused. Therefore, these conditions are studied and treated as if they do not have any common mechanisms or underlying basis. However, that may not be the case and the concept that the various post-menopausal conditions are all related to reproductive functioning and thus may have commonalities in mechanisms has been advanced [1].

**Figure 1:**
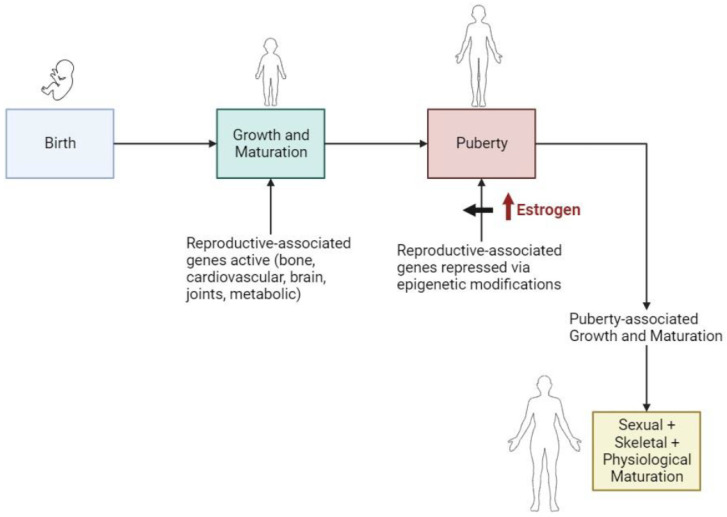
Sequence of epigenetic modifications associated with onset of puberty and progression to skeletal and physiological maturity without intervening pregnancy. Epigenetic modifications repress genes that are associated with pregnancy and lactation which allows for post-puberty growth and maturation. Epigenetic repression maintained by menstrual cycles.

Building on that commonality concept and the discussion contained earlier in this review, the hypothesis that the commonality [1] for post-menopausal conditions/diseases may be based on a system of reversible epigenetic signatures that are influenced by sex hormones at puberty and then during pregnancy and lactation, and, finally, by menopause is advanced. As outlined in Figure 1, during the early pre-puberty years, many genes associated with the relevant tissues are either not epigenetically modified to accommodate initial growth and maturation or are modified in a specific manner. However, at puberty, many of them are now epigenetically modified to be in a suppressed or quiescent state in preparation for reproductive functioning, and potentially some of them being active may not be compatible with the adolescent growth and maturation sequences. In this scenario, the targeted genes are maintained in an epigenetically impacted state by the recurrent monthly menstrual cycles until skeletal maturity is obtained and thereafter.

**Figure 2:**
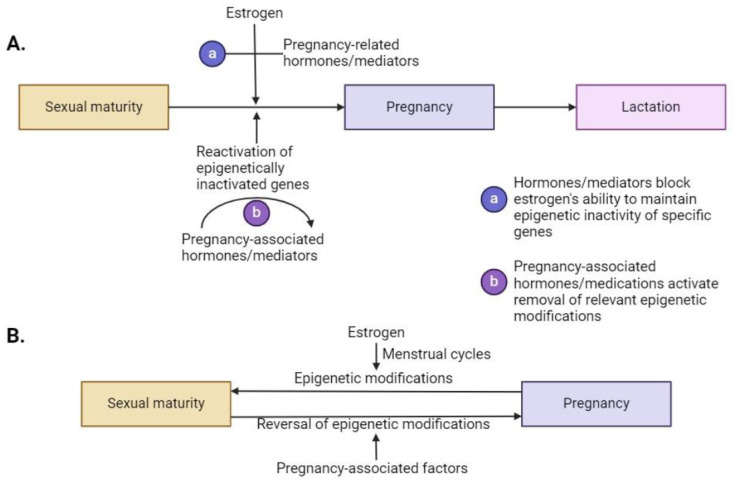
Pregnancy leads to the re-activation of genes influenced by puberty-associated epigenetic modifications allowing for essential activities leading to a successful pregnancy followed by lactation. (**A**) Pregnancy-related factors either negate the effects of estrogen (and potentially other sex hormones like progesterone) to maintain the epigenetic modifications, or pregnancy-related factors override the effects of estrogen via enzymes that impact the epigenetic modifications. (**B**) Following cessation of pregnancy and lactation, menstrual cycles re-establish the epigenetic modifications that again lead to repression of the pregnancy-associated genes until the next pregnancy. With frequent pregnancies, the ability of menstrual cycles to re-establish a homeostatic state with regard to these affected genes may be compromised.

However, at the time of pregnancy, these epigenetically repressed genes are likely required for a successful pregnancy and, thus, the epigenetic repression is reversed by mechanisms and enzymes that override the influence of estrogen/progesterone, leading to the involvement of systems (e.g., calcium mobilization from bone, adapting to stresses on the cardiovascular system, metabolic needs for nutrition, and brain center involvement) that benefit fetal growth and the health of the mother. This hypothetical process is outlined in Figure 2 (Panel A). In this scheme, epigenetically repressed genes required for a successful pregnancy that were epigenetically modified as a result of puberty become reactivated for pregnancy, with additional changes potentially reversed for lactation. Once pregnancy and lactation are completed, the re-institution of menstrual cycles leads to epigenetic re-repression of the involved genes. Such cycles of epigenetic repression and then de-repression would occur with each pregnancy, creating an equilibrium between the two scenarios (Figure 2, Panel B). Multiple pregnancies without sufficient time between cycles may lead to incomplete re-repression resulting in risks elaborated following early menopause. Some reports have indicated that parity is associated with increased risk for CVD [167,168,169], dementia [170], metabolic disease [171], and knee OA [172,173], but not fracture risk due to OP [21]. Therefore, with increased parity, there may be incomplete re-establishment of a repressed state by menstrual cycles.

**Figure 3:**
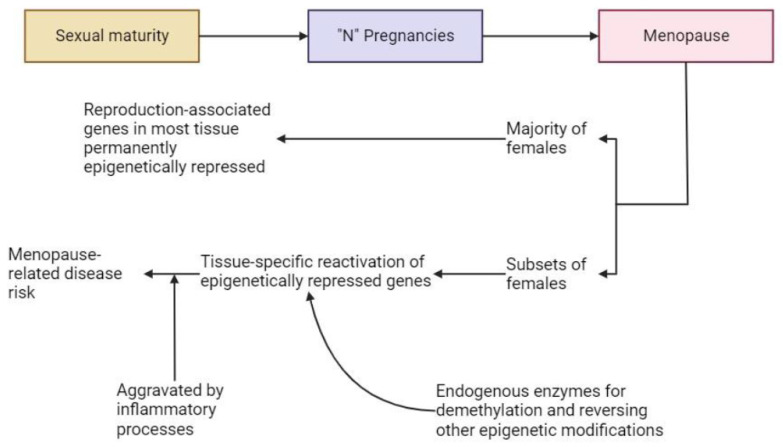
Possible role of tissue-specific reversal of puberty-associated genes to become re-activated following menopause. Following menopause and the loss of estrogen to maintain the epigenetically repressed genes, through either genetic variables or other unknown factors, the pregnancy-associated genes become re-activated by tissue-specific endogenous enzymes leading to the spectrum of menopause-associated conditions/diseases. Not all females would have all of the disease risks. Once initiated, the impact of the de-repression could be aggravated by ongoing inflammatory processes. The cells central to this proposed scheme may be the endothelial cells of the microvasculature in a tissue-specific manner (discussed in more detail in Section 7 of this review).

Following menopause, which is a process that can take years to complete and is not an acute event, some tissues may de-repress the estrogen-driven epigenetic modifications in the absence of pregnancy due to genetic factors or environmental factors [Figure 3]. In this scenario, different tissues involved in the pregnancy/lactation-associated processes may become active by removing some of the epigenetic modifications, leading to chronic and unregulated expression of genes and gene products contributing to post-menopausal diseases and disease risks. Not all females would be at equal risk for such conditions, and not all affected females would have all of the conditions/diseases, as the modifications would be tissue-specific and not general in this construct [Figure 3].

The investigation of this construct to explain the spectrum of post-menopausal diseases and conditions could remove it from the study of each condition/disease individually, focus research on the commonalities of the mechanisms involved, and address the spectrum of conditions as components of a system rather than a collection of disparate diseases.

## 7. What Cells May Be Involved in the Relevant Epigenetic Modifications Outlined in Section 6?

The conditions/diseases associated with the post-menopausal state involve a variety of tissues ranging from the brain (dementia), bone (osteoporosis), adipose tissue (obesity), the cardiovascular system (heart and vascular disease risk), and joint tissues (osteoarthritis). Thus, there is a spectrum of mechanisms that could be involved, and these include the following: (1) the cells unique to each tissue are the primary target for the epigenetic modifications; (2) as all of the tissues involved are vascularized, the primary target could be the endothelial cells in the microvasculature of each tissue; and (3) as all of the tissues involved are also innervated (or are brain centers), the primary effectors relate to the neuronal cells that then either regulate the endothelial cells (neurovascular regulation) or the target cells directly. For the latter, this may involve the brain centers affecting each tissue. The first option, that tissue-specific target cells are the targets of the epigenetically modified gene expression profile, is the most difficult to envision, as bone is widely distributed in the body and exists in a variety of mechanical and biological environments, conditions that may make it hard to target all of the cells equally or completely. Adipose tissue is also widely distributed, but some tissues could be affected more than others leading to a selective influence on specific adipose tissues. Regarding dementia, specific areas of the brain could be involved, and this post-menopausal condition could result from epigenetic modification of cells localized to that specific type of tissue. Thus, “option 1” could be in play regarding the post-menopausal conditions that are epigenetically dependent, but if this option were the dominant mechanism, there may be challenges to implementing this mechanism given the diversity of some of the tissues.

All of the tissues involved in the post-menopausal conditions/diseases are vascularized except articular cartilage in OA and that condition is likely due to alterations in the other tissues that are involved in joint functioning and the cartilage is only the “weak link” in the disease process (discussed in [10]). While all of the relevant tissues are vascularized, it is likely that the endothelial cells in the different tissues have differentiated to effectively function in those tissues in a paracrine manner (reviewed in [112,174,175,176]; discussed in [113,114]). Such tissue-specific characteristics appear to be lost when endothelial cells are cultured in vitro [177] but also appear to utilize epigenetic modifications in the regulation of their function in specific environments [178]. Thus, a commonality in post-menopausal conditions could be the targeting of endothelial cells in the different relevant tissue environments to alter their functioning via epigenetic modification, leading to tissue-specific disease risk after menopause by disrupting the integrity of the paracrine system involving the endothelial cells and their target cells. As it is known that microvascular endothelial cells in some tissues are functionally altered during pregnancy in preclinical models [179,180], there is precedent for endothelial cells to be altered by this condition. However, as rabbits are induced ovulators, they do not have menstrual cycles as do humans, so the models have some limitations regarding the further exploration of the concept discussed.

Further evidence for the potential role of endothelial cells of the microvasculature in post-menopausal conditions can be derived from studies indicating that exercise can prevent or alleviate the progression of bone loss and conditions such as osteoporosis [181,182,183,184], can prevent the loss of cardiovascular integrity [185,186,187,188], osteoarthritis [189], and dementia [190,191,192,193], as well as the prevention of aging-related endothelial cell senescence [194,195,196,197]. In contrast, physical inactivity can contribute to brain-associated changes and such changes are mediated in part by epigenetic alterations [198]. Physical activity has also been reported to potentially prevent loss of cognition and function via enhanced hippocampus neurogenesis in a variety of species [199]. Furthermore, some of the age-related risk for diseases is mediated by epigenetic modifications [200], and these can be influenced by exercise in systems such as the cardiovascular system [185,201]. Also of relevance to this discussion are reviews that address the issue of endothelial cell senescence on the development and progression of dementias such as Alzheimer’s disease [202]. Finally, and relevant to the present discussion, are reports that exercise can impact the cognitive function of older females more than males, and this appears to be related to parity in females [203,204].

Based on the above discussion, the question then arises as to how exercise (e.g., mechanical loading) could influence endothelial cell epigenetics directly. Endothelial cells express the sensor of mechanical loading piezo1 [205] and this senses fluid flow [206]. Piezo1-dependent regulation of brain vascular development has been reported [207,208]. Relevant to the current discussion of the role of endothelial cells and epigenetics in post-menopausal conditions/diseases, mechanical stimulation of endothelial cells leads to epigenetic modifications [209], changes that could influence the interaction of the endothelial cells in specific tissues. However, it has not yet been reported as to whether mechanical loading of endothelial cells from different vascular and tissue environments responds similarly or differently.

Alternatively, some vascular functioning can be mediated by the associated neuro-elements that often parallel the endothelial cells in the microcirculation. As mentioned previously, aspects of this relationship appear to be modified during pregnancy in the rabbit model [179,180]. Thus, neuro-regulation of differentiated endothelial cells in specific tissues could exert tissue-specific responses and potentially contribute to endothelial responses occurring after the decline in sex hormone expression following menopause. Thus, both neuro-regulation and exercise appear to impact vaso-regulation, likely in part through the regulation of endothelial cells. Quite possibly this is not an “either or” situation and mechanical modulation of endothelial cells and neuro-regulation of the cells could work together or exert different levels of influence in different tissues. Some of these possibilities will need to be addressed by future research.

## 8. Conclusions and the Way Forward

While the rationale for the bone-associated variables being linked to pregnancy and lactation and then the development of osteoporosis is fairly well developed, how the dementia risk in the post-menopausal state relates to involvement in reproductive activities still remains to be developed. Why only a subset of post-menopausal females develop dementia and why specific areas of the brain may be affected versus others, also remains to be determined. However, in both OP and dementia, inflammatory processes appear to be involved in exacerbating the disease progression.

Given the influence of parity and early menopause on development of post-menopausal diseases and conditions, one could postulate that epigenetic processes are involved and the reason that only a subset of post-menopausal females are affected by conditions such as OP and AD is due to a combination of genetic and epigenetic variables, with the epigenetic modifications requiring longer exposure to menstrual cycles following pregnancy/lactation-associated intervals to maintain the relevant cells in a repressed state via those genes activated during pregnancy and lactation. As Alzheimer’s disease usually develops long after the menopause transition in most individuals (i.e., >20 years), it is challenging to comprehend how such a time course after menopause could translate to an impact on the at-risk females during a 9-month pregnancy and perhaps a 1-year lactation.

While it is still not yet clear how the various conditions, disease risks, and diseases arising after menopause occur at the mechanistic level, the concept that it involves the microvasculature and tissue-specific endothelial cells was advanced and should be the focus of future investigations. The possible use in the future of drugs or interventions capable of modifying epigenetic alterations contributing to post-menopausal conditions/diseases in a controlled manner (e.g., removal, addition) may be a feasible avenue for research going forward. Such drugs have been proposed to be applied in conditions affecting bone [210], cancer [211,212], neurodegenerative disorders [213,214], cardiovascular conditions [215,216], and healthy aging [217]. The development of such “epi-drugs” is not without challenges [218], particularly since such interventions will have to be personalized for post-menopausal conditions that arise in different tissues, may not result from a single phenotypic pattern, and may occur in conjunction with multiple co-morbidities as the individual ages.

As the epi-drug approach may involve tissue-specific epigenetic modifications and their reversal/addition via a variety of mechanisms (e.g., DNA modification, histone modification, and others), this will be challenging to perform with heterogeneous human/patient populations. In the meantime, perhaps one of the most effective non-drug interventions that are beneficial for human functioning across the lifespan is exercise protocols [219]. While likely effective across the lifespan, they may be particularly effective in mid-life and beyond when mobility issues and co-morbidities may inhibit motivation for exercise in post-menopausal females.

Finally, another non-drug approach could be the use of specific diets to interfere with or deter the development of post-menopausal conditions. Of particular interest has been the study of Mediterranean diets or diets rich in soy isoflavonoids/flavones [220,221,222,223] and their effects on CVD, dementia, and metabolic disease in post-menopausal females. One component of such diets is genistein, a soybean isoflavone [224], and a molecule that can exert its influence via ER-beta, as well as has effects on endothelial cells [225,226,227,228,229]. A clinical trial of genistein alone has been reported to have a beneficial effect on post-menopausal females with metabolic syndrome [230]. Relevant to the present discussion are also reports that flavonoids, including genistein, can exert epigenetic effects [231,232,233,234,235]. Therefore, several aspects of these diets and natural products are very compatible with the proposed epigenetic-based mechanism for post-menopausal conditions. 

While many of the diet-related benefits in post-menopausal females are modest [221,222], going forward, a focus of research should be the combination of diets such as the Mediterranean diet or those containing soy-derived flavonoids and related molecules with validated exercise programs. The combination of these two “natural” interventions could lead to additive or synergistic effects on the development and progression of post-menopausal conditions without the need for synthetic pharmaceuticals. Such a combination of interventions could also be initiated prior to the onset of menopause in a proactive manner and thus be preventative.

## Data Availability

Not applicable.

## References

[1] Hart D.A. (2022). Sex Differences in Biological Systems and the Conundrum of Menopause: Potential Commonalities in Post-Menopausal Disease Mechanisms. Int. J. Mol. Sci..

[2] Hart D.A. (2023). Sex differences in musculoskeletal injury and disease risks across the lifespan: Are there unique subsets of females at higher risk than males for these conditions at distinct stages of the life cycle?. Front. Physiol..

[3] Harrod C.G., Bendok B.R., Batjer H.H. (2005). Interactions between melatonin and estrogen may regulate cerebrovascular function in women: Clinical implications for the effective use of HRT during menopause and aging. Med. Hypotheses.

[4] Gannon O., Robison L., Custozzo A., Zuloaga K. (2018). Sex differences in risk factors for vascular contributions to cognitive impairment & dementia. Neurochem. Int..

[5] Rehman H.U., Masson E.A. (2005). Neuroendocrinology of female aging. Gend. Med..

[6] Yoo J.E., Shin D.W., Han K., Kim D., Won H., Lee J., Kim S., Nam G.E., Park H.S. (2020). Female reproductive factors and the risk of dementia: A nationwide cohort study. Eur. J. Neurol..

[7] Gannon O.J., Naik J.S., Riccio D., Mansour F.M., Abi-Ghanem C., Salinero A.E., Kelly R.D., Brooks H.L., Zuloaga K.L. (2023). Menopause causes metabolic and cognitive impairments in a chronic cerebral hypoperfusion model of vascular contributions to cognitive impairment and dementia. Biol. Sex Differ..

[8] Hogervorst E., Temple S., O’Donnell E. (2023). Sex differences in dementia. Curr. Top. Behav. Neurosci..

[9] Szoeke C., Downie S., Parker A., Phillips S. (2021). Sex hormones, vascular factors and cognition. Front. Neuroendocr..

[10] Hart D.A. (2022). Osteoarthritis as an Umbrella Term for Different Subsets of Humans Undergoing Joint Degeneration: The Need to Address the Differences to Develop Effective Conservative Treatments and Prevention Strategies. Int. J. Mol. Sci..

[11] Hart D.A. (2023). Regulation of Bone by Mechanical Loading, Sex Hormones, and Nerves: Integration of Such Regulatory Complexity and Implications for Bone Loss during Space Flight and Post-Menopausal Osteoporosis. Biomolecules.

[12] Fenton A., Smart C., Goldschmidt L., Price V., Scott J. (2023). Fat mass, weight and body shape changes at menopause—Causes and consequences: A narrative review. Climacteric.

[13] Speth R.C., D’ambra M., Ji H., Sandberg K. (2018). A heartfelt message, estrogen replacement therapy: Use it or lose it. Am. J. Physiol. Circ. Physiol..

[14] Kondapalli A.V., Kamanda-Kosseh M., Williams J.M., Shiau S., Bucovsky M., Colon I., Shane E., Cohen A. (2023). Clinical characteristics of pregnancy and lactation associated osteoporosis: An online survey study. Osteoporos. Int..

[15] Samelson E.J., Hannan M.T. (2006). Epidemiology of osteoporosis. Curr. Rheumatol. Rep..

[16] Hart D.A. (2023). Homo sapiens—A species not designed for space flight: Health risks in low Earth orbit and beyond, including potential risks when traveling beyond the geomagnetic field of Earth. Life.

[17] Parker S.E., Troisi R., Wise L.A., Palmer J.R., Titus-Ernstoff L., Strohsnitter W.C., Hatch E.E. (2014). Menarche, Menopause, Years of Menstruation, and the Incidence of Osteoporosis: The Influence of Prenatal Exposure to Diethylstilbestrol. J. Clin. Endocrinol. Metab..

[18] Rai S.K., Shaki O., Gupta T.P., Upreti V., Patil D. (2021). Does parity and duration of lactation have any effect on the bone mineral density of the femur and lumbar spine in Indian women? A cross-sectional study from the Northeast region of India. J. Fam. Med. Prim. Care.

[19] Seo E., Lee Y., Kim H.C. (2021). Association between Parity and Low Bone Density among Postmenopausal Korean Women. J. Prev. Med. Public Health.

[20] Yang Y., Wang S., Cong H. (2022). Association between parity and bone mineral density in postmenopausal women. BMC Women’s Health.

[21] de Bakker C.M.J., Burt L.A., Gabel L., Hanley D.A., Boyd S.K. (2019). Associations between Breastfeeding History and Early Postmenopausal Bone Loss. Calcif. Tissue Int..

[22] Sochocka M., Karska J., Pszczołowska M., Ochnik M., Fułek M., Fułek K., Kurpas D., Chojdak-Łukasiewicz J., Rosner-Tenerowicz A., Leszek J. (2023). Cognitive Decline in Early and Premature Menopause. Int. J. Mol. Sci..

[23] Coughlan G.T., Coughlan G.T., Betthauser T.J., Betthauser T.J., Boyle R., Boyle R., Koscik R.L., Koscik R.L., Klinger H.M., Klinger H.M. (2023). Association of Age at Menopause and Hormone Therapy Use with Tau and β-Amyloid Positron Emission Tomography. JAMA Neurol..

[24] Karamitrou E.K., Anagnostis P., Vaitsi K., Athanasiadis L., Goulis D.G. (2023). Early menopause and premature ovarian insufficiency are associated with increased risk of dementia: A systematic review and meta-analysis of observational studies. Maturitas.

[25] Cezarino P.Y.A., Simoes R.D.S., Baracat E.C., Junior J.M.S. (2018). Are women living with HIV prone to osteoporosis in post-menopause? A systematic review. Rev. Assoc. Med. Bra. (1992).

[26] Gómez-Vaquero C., Hernández J.L., Olmos J.M., Cerdà D., Calleja C.H., López J.A.M., Arboleya L., del Rey F.J.A., Pardo S.M., Vilamajó I.R. (2023). High incidence of clinical fragility fractures in postmenopausal women with rheumatoid arthritis. A case-control study. Bone.

[27] Shen L., Lv J., Li J., Zhou J., Wang X. (2023). Managing osteoporosis in COPD. Endocr. Metab. Immune Disord.-Drug Targets.

[28] Wu W., Li X., Di J., Zhou H., Niu H., Chen L., Sha Q., Yang M. (2023). The relationship between dietary inflammatory index and bone mineral density in CKD patients. Ther. Apher. Dial..

[29] Gil-Montoya J.A., Garrido-Martínez M., Barrios-Rodríguez R., Ramos-García P., Lenouvel D., Montes-Castillo C., Martínez-Ramírez M.J. (2020). Association between low bone mineral density and periodontitis in generally healthy perimenopausal women. J. Periodontol..

[30] Torres H.M., Arnold K.M., Oviedo M., Westendorf J.J., Weaver S.R. (2023). Inflammatory Processes Affecting Bone Health and Repair. Curr. Osteoporos. Rep..

[31] Franceschi C., Campisi J. (2014). Chronic Inflammation (Inflammaging) and Its Potential Contribution to Age-Associated Diseases. J. Gerontol. A Ser. Biol. Sci. Med. Sci..

[32] Yigitol I., Gulumsek E., Ozturk H.A., Arici F.N., Akbal K., Pirinci O., Karacay M., Cihan T.N., Totik Z.G., Akyildiz M.A. (2023). Serum periostin levels are significantly higher in patients with primary hyperthyroidism and closely related to os-teoporosis. Exp. Clin. Endocrinol. Diabetes.

[33] Chen Z., Lv M., Liang J., Yang K., Li F., Zhou Z., Qiu M., Chen H., Cai Z., Cui W. (2023). Neuropeptide Y-Mediated Gut Microbiota Alterations Aggravate Postmenopausal Osteoporosis. Adv. Sci..

[34] Ahire J.J., Kumar V., Rohilla A. (2023). Understanding Osteoporosis: Human Bone Density, Genetic Mechanisms, Gut Microbiota, and Future Prospects. Probiotics Antimicrob. Proteins.

[35] Yan Y., Chen W., Wang J., Huang J., Lv J., Zhao H., Guo L. (2020). Serum anti-Müllerian hormone levels are associated with low bone mineral density in premenopausal women. Biomarkers.

[36] Karlamangla A.S., Shieh A., A Greendale G., Yu E.W., Burnett-Bowie S.-A.M., Sluss P.M., Martin D., Morrison A., Finkelstein J.S. (2020). Anti-Mullerian Hormone as Predictor of Future and Ongoing Bone Loss During the Menopause Transition. J. Bone Miner. Res..

[37] Abrahamsen B., Madsen J.S., Tofteng C.L., Stilgren L., Bladbjerg E.M., Kristensen S.R., Brixen K., Mosekilde L. (2003). A Common Methylenetetrahydrofolate Reductase (C677T) Polymorphism Is Associated with Low Bone Mineral Density and Increased Fracture Incidence after Menopause: Longitudinal Data from the Danish Osteoporosis Prevention Study. J. Bone Miner. Res..

[38] Catheline S.E., Kaiser E., Eliseev R.A. (2023). Mitochondrial Genetics and Function as Determinants of Bone Phenotype and Aging. Curr. Osteoporos. Rep..

[39] Himič V., Syrmos N., Ligarotti G.K.I., Kato S., Fehlings M.G., Ganau M. (2023). The role of genetic and epigenetic factors in determining the risk of spinal fragility fractures: New insights in the management of spinal osteoporosis. Quant. Imaging Med. Surg..

[40] Zhao R., Chen Y., Wang D., Zhang C., Song H., Ni G. (2023). Role of irisin in bone diseases. Front. Endocrinol..

[41] Cheng C.-Y. (2023). Risk of osteoporosis among individuals with varicose veins: A multi-institution cohort study. Arch. Osteoporos..

[42] Mishra P., Davies D.A., Albensi B.C. (2022). The Interaction between NF-κB and Estrogen in Alzheimer’s Disease. Mol. Neurobiol..

[43] Au A., Feher A., McPhee L., Jessa A., Oh S., Einstein G. (2016). Estrogens, inflammation and cognition. Front. Neuroendocr..

[44] Skoczek-Rubińska A., Muzsik-Kazimierska A., Chmurzynska A., Jamka M., Walkowiak J., Bajerska J. (2021). Inflammatory Potential of Diet Is Associated with Biomarkers Levels of Inflammation and Cognitive Function among Postmenopausal Women. Nutrients.

[45] Robison L.S., Gannon O.J., Salinero A.E., Zuloaga K.L. (2018). Contributions of sex to cerebrovascular function and pathology. Brain Res..

[46] Rizzo M.R., Fasano R., Paolisso G. (2020). Adiponectin and Cognitive Decline. Int. J. Mol. Sci..

[47] Fan Y.-G., Pang Z.-Q., Wu T.-Y., Zhang Y.-H., Xuan W.-Q., Wang Z., Yu X., Li Y.-C., Guo C., Wang Z.-Y. (2020). Vitamin D deficiency exacerbates Alzheimer-like pathologies by reducing antioxidant capacity. Free Radic. Biol. Med..

[48] McCollum L.E., Das S.R., Xie L., de Flores R., Wang J., Xie S.X., Wisse L.E., Yushkevich P.A., Wolk D.A. (2021). Oh brother, where art tau? Amyloid, neurodegeneration, and cognitive decline without elevated tau. NeuroImage Clin..

[49] Knopman D.S., Hershey L. (2023). Implications of the Approval of Lecanemab for Alzheimer Disease Patient Care. Neurology.

[50] Hussain S.M., Wang Y., Giles G.G., Graves S., Wluka A.E., Cicuttini F.M. (2018). Female Reproductive and Hormonal Factors and Incidence of Primary Total Knee Arthroplasty Due to Osteoarthritis. Arthritis Rheumatol..

[51] De Roover A., Escribano-Núñez A., Monteagudo S., Lories R. (2023). Fundamentals of osteoarthritis: Inflammatory mediators in osteoarthritis. Osteoarthr. Cartil..

[52] Horváth E., Sólyom Á., Székely J., Nagy E.E., Popoviciu H. (2023). Inflammatory and Metabolic Signaling Interfaces of the Hypertrophic and Senescent Chondrocyte Phenotypes Associated with Osteoarthritis. Int. J. Mol. Sci..

[53] Prather H., Cheng J. (2023). Relationship of Chronic Systemic Inflammation to Both Chronic Lifestyle-Related Diseases and Osteoarthritis: The Case for Lifestyle Medicine for Osteoarthritis. HSS J..

[54] Qadri M.M. (2023). Targeting CD44 Receptor Pathways in Degenerative Joint Diseases: Involvement of Proteoglycan-4 (PRG4). Pharmaceuticals.

[55] Heard B.J., Barton K.I., Chung M., Achari Y., Shrive N.G., Frank C.B., Hart D.A. (2015). Single intra-articular dexamethasone injection immediately post-surgery in a rabbit model mitigates early inflammatory responses and post-traumatic osteoarthritis-like alterations. J. Orthop. Res..

[56] Heard B.J., Barton K.I., Abubacker S., Chung M., Martin C.R., Schmidt T.A., Shrive N.G., Hart D.A. (2021). Synovial and cartilage responsiveness to peri-operative hyaluronic acid ± dexamethasone administration following a limited injury to the rabbit stifle joint. J. Orthop. Res..

[57] Barton K.I., Heard B.J., Sevick J.L., Martin C.R., Shekarforoush S.M., Chung M., Achari Y., Frank C.B., Shrive N.G., Hart D.A. (2018). Posttraumatic Osteoarthritis Development and Progression in an Ovine Model of Partial Anterior Cruciate Ligament Transection and Effect of Repeated Intra-articular Methylprednisolone Acetate Injections on Early Disease. Am. J. Sports Med..

[58] Shumnalieva R., Kotov G., Monov S. (2023). Obesity-Related Knee Osteoarthritis—Current Concepts. Life.

[59] Lynskey S.J., Macaluso M.J., Gill S.D., McGee S.L., Page R.S. (2023). Biomarkers of Osteoarthritis—A Narrative Review on Causal Links with Metabolic Syndrome. Life.

[60] Jiménez-Muro M., Soriano-Romaní L., Mora G., Ricciardelli D., Nieto J.A. (2023). The microbiota-metabolic syndrome axis as a promoter of metabolic osteoarthritis. Life Sci..

[61] Ramirez M.F., Honigberg M., Wang D., Parekh J.K., Bielawski K., Courchesne P., Larson M.D., Levy D., Murabito J.M., Ho J.E. (2023). Protein Biomarkers of Early Menopause and Incident Cardiovascular Disease. J. Am. Hear. Assoc..

[62] Isola J.V.V., Ko S., Ocañas S.R., Stout M.B. (2023). Role of Estrogen Receptor α in Aging and Chronic Disease. Adv. Geriatr. Med. Res..

[63] Lee S.-R., Directo D. (2023). Fish Oil Supplementation with Resistance Exercise Training Enhances Physical Function and Cardiometabolic Health in Postmenopausal Women. Nutrients.

[64] Son W.-H., Park H.-T., Jeon B.H., Ha M.-S. (2023). Moderate intensity walking exercises reduce the body mass index and vascular inflammatory factors in postmenopausal women with obesity: A randomized controlled trial. Sci. Rep..

[65] Kottilil S., Mathur P. (2022). The influence of inflammation on cardiovascular disease in women. Front. Glob. Women’s Health.

[66] Klein G.L. (2022). Is calcium a link between inflammatory bone resorption and heart disease?. eLife.

[67] Mansanguan C., Mansanguan C., Maneerat Y., Maneerat Y. (2022). *PPBP* gene as a biomarker for coronary heart disease risk in postmenopausal Thai women. PeerJ.

[68] Justina V.D., Miguez J.S.G., Priviero F., Sullivan J.C., Giachini F.R., Webb R.C. (2021). Sex Differences in Molecular Mechanisms of Cardiovascular Aging. Front. Aging.

[69] Barris C.T., Faulkner J.L., de Chantemèle E.J.B. (2023). Salt Sensitivity of Blood Pressure in Women. Hypertension.

[70] Bedeković D., Bošnjak I., Šarić S., Kirner D., Novak S. (2023). Role of Inflammatory Cytokines in Rheumatoid Arthritis and Development of Atherosclerosis: A Review. Medicina.

[71] Mattay S.S., Zamani M., Saturno D., Loftus E.V., Ciorba M.A., Yarur A., Singh S., Deepak P. (2023). Risk of Major Adverse Cardiovascular Events in Immune-Mediated Inflammatory Disorders on Biologics and Small Molecules: Network Meta-Analysis. Clin. Gastroenterol. Hepatol..

[72] Schattner A. (2023). The Cardiovascular Burden of Rheumatoid Arthritis—Implications for Treatment. Am. J. Med..

[73] Benlidayi I.C. (2023). Exercise therapy for improving cardiovascular health in rheumatoid arthritis. Rheumatol. Int..

[74] Lahita R.G. (1990). Sex hormones and the immune system—Part 1. Hum. Data.

[75] Mohammadisima N., Farshbaf-Khalili A., Ostadrahimi A., Pourmoradian S. (2022). Positive relation between dietary inflammatory index and osteoporosis in postmenopausal women. Int. J. Vitam. Nutr. Res..

[76] Dong W., Peng Q., Liu Z., Xie Z., Guo X., Li Y., Chen C. (2023). Estrogen plays an important role by influencing the NLRP3 inflammasome. Biomed. Pharmacother..

[77] Zhang X., Huangfu Z., Wang S. (2023). Review of mendelian randomization studies on age at natural menopause. Front. Endocrinol..

[78] Portela A., Esteller M. (2010). Epigenetic modifications and human disease. Nat. Biotechnol..

[79] Mirisola M.G. (2023). The Nutriepigenome. Genes.

[80] Qiu L., Jing Q., Li Y., Han J. (2023). RNA modification: Mechanisms and therapeutic targets. Mol. Biomed..

[81] la Torre A., Vecchio F.L., Greco A. (2023). Epigenetic Mechanisms of Aging and Aging-Associated Diseases. Cells.

[82] De La Cruz B.M., Darsinou M., Riccio A. (2023). From form to function: m6A methylation links mRNA structure to metabolism. Adv. Biol. Regul..

[83] Huang M., Guo J., Liu L., Jin H., Chen X., Zou J. (2023). m6A demethylase FTO and osteoporosis: Potential therapeutic interventions. Front. Cell Dev. Biol..

[84] Gu Y., Song Y., Pan Y., Liu J. (2024). The essential roles of m6A modification in osteogenesis and common bone diseases. Genes Dis..

[85] Hernández-Oliveras A., Zarain-Herzberg A. (2024). The role of Ca^2+^-signaling in the regulation of epigenetic mechanisms. Cell Calcium.

[86] Mao Y., Zhao K., Chen N., Fu Q., Zhou Y., Kong C., Li P., Yang C. (2023). A 2-decade bibliometric analysis of epigenetics of cardiovascular disease: From past to present. Clin. Epigenetics.

[87] Sum H., Brewer A.C. (2023). Epigenetic modifications as therapeutic targets in atherosclerosis: A focus on DNA methylation and non-coding RNAs. Front. Cardiovasc. Med..

[88] Kiełbowski K., Herian M., Bakinowska E., Banach B., Sroczyński T., Pawlik A. (2023). The Role of Genetics and Epigenetic Regulation in the Pathogenesis of Osteoarthritis. Int. J. Mol. Sci..

[89] Cheng C., Wu Y., Huang Y., Xue Q., Wang Y., Liao F., Wang X., Miao C. (2023). Epigenetic modification and exosome effects on autophagy in osteoarthritis. Biochem. Pharmacol..

[90] Tan D., Huang Z., Zhao Z., Chen X., Liu J., Wang D., Deng Z., Li W. (2023). Single-cell sequencing, genetics, and epigenetics reveal mesenchymal stem cell senescence in osteoarthritis (Review). Int. J. Mol. Med..

[91] Qureshi I.A., Mehler M.F. (2010). Genetic and epigenetic underpinnings of sex differences in the brain and in neurological and psychiatric disease susceptibility. Prog. Brain Res..

[92] Machado-Vieira R., Ibrahim L., Zarate C.A. (2011). Histone deacetylases and mood disorders: Epigenetic programming in gene-environment interactions. CNS Neurosci. Ther..

[93] Shao N., Ye T., Xuan W., Zhang M., Chen Q., Liu J., Zhou P., Song H., Cai B. (2023). The effects of N6-methyladenosine RNA methylation on the nervous system. Mol. Cell. Biochem..

[94] Wang H., Guo B., Guo X. (2023). Histone demethylases in neurodevelopmet and neurodegenerative diseases. Int. J. Neurosci..

[95] Smith E.N.L., Chandanathil M., Millis R.M. (2023). Epigenetic Mechanisms in Obesity: Broadening Our Understanding of the Disease. Cureus.

[96] Trang K., Grant S.F. (2023). Genetics and epigenetics in the obesity phenotyping scenario. Rev. Endocr. Metab. Disord..

[97] Calabriso N., Massaro M., Scoditti E., Carluccio C., Verni T., Carluccio M.A. (2023). Epigenetic mechanisms in vascular in-flammation: Modulation of endothelial adhesion molecules and endothelium-leukocyte adhesion. Front. Biosci. (Landmark Ed.).

[98] Bacon E.R., Brinton R.D. (2021). Epigenetics of the developing and aging brain: Mechanisms that regulate onset and outcomes of brain reorganization. Neurosci. Biobehav. Rev..

[99] Bacon E.R., Mishra A., Wang Y., Desai M.K., Yin F., Brinton R.D. (2018). Neuroendocrine aging precedes perimenopause and is regulated by DNA methylation. Neurobiol. Aging.

[100] Knight A.K., Spencer J.B., Smith A.K. (2024). DNA methylation as a window into female reproductive aging. Epigenomics.

[101] Pérez-Cremades D., Mompeón A., Vidal-Gómez X., Hermenegildo C., Novella S. (2018). miRNA as a New Regulatory Mechanism of Estrogen Vascular Action. Int. J. Mol. Sci..

[102] Bjune J.-I., Strømland P.P., Jersin R., Mellgren G., Dankel S.N. (2022). Metabolic and Epigenetic Regulation by Estrogen in Adipocytes. Front. Endocrinol..

[103] Shepherd R., Bretherton I., Pang K., Mansell T., Czajko A., Kim B., Vlahos A., Zajac J.D., Saffery R., Cheung A. (2022). Gender-affirming hormone therapy induces specific DNA methylation changes in blood. Clin. Epigenetics.

[104] Gratton D.R., Ladyman S.R. (2020). Neurophysicological and cognitive changes in pregnancy. Handb. Clin. Neurol..

[105] Gutaj P., Sibiak R., Jankowski M., Awdi K., Bryl R., Mozdziak P., Kempisty B., Wender-Ozegowska E. (2020). The Role of the Adipokines in the Most Common Gestational Complications. Int. J. Mol. Sci..

[106] Gomes P.R.L., Motta-Teixeira L.C., Gallo C.C., Buonfiglio D.D.C., de Camargo L.S., Quintela T., Reiter R.J., Amaral F.G.D., Cipolla-Neto J. (2020). Maternal pineal melatonin in gestation and lactation physiology, and in fetal development and programming. Gen. Comp. Endocrinol..

[107] Carvalho D.P., Dias A.F., Sferruzzi-Perri A.N., Ortiga-Carvalho T.M. (2022). Gaps in the knowledge of thyroid hormones and placental biology. Biol. Reprod..

[108] Rassie K., Giri R., Joham A.E., Teede H., Mousa A. (2023). Prolactin in Pregnancies Affected by Pre-Existing Maternal Metabolic Conditions: A Systematic Review. Int. J. Mol. Sci..

[109] Iosim S., MacKay M., Westover C., E Mason C. (2019). Translating current biomedical therapies for long duration, deep space missions. Precis. Clin. Med..

[110] Park S.-K., Stefanyshyn D.J., Ramage B., A Hart D., Ronsky J.L. (2009). Relationship between knee joint laxity and knee joint mechanics during the menstrual cycle. Br. J. Sports Med..

[111] Park S.-K., Stefanyshyn D.J., Loitz-Ramage B., Hart D.A., Ronsky J.L. (2009). Changing Hormone Levels during the Menstrual Cycle Affect Knee Laxity and Stiffness in Healthy Female Subjects. Am. J. Sports Med..

[112] Augustin H.G., Koh G.Y. (2017). Organotypic vasculature: From descriptive heterogeneity to functional pathophysiology. Science.

[113] Hart D.A. (2023). Are secondary effects of bisphosphonates on the vascular system of bone contributing to increased risk for atypical femoral fractures in osteoporosis?. BioEssays.

[114] Hart D.A. (2023). Towards understanding how bisphosphonate-dependent alterations to nutrient canal integrity can contribute to risk for atypical femoral fractures: Biomechanical considerations and potential relationship to a real-world analogy. BioEssays.

[115] Kanherkar R.R., Bhatia-Dey N., Csoka A.B. (2014). Epigenetics across the human lifespan. Front. Cell Dev. Biol..

[116] Bar-Sadeh B., Rudnizky S., Pnueli L., Bentley G.R., Stöger R., Kaplan A., Melamed P. (2020). Unravelling the role of epigenetics in reproductive adaptations to early-life environment. Nat. Rev. Endocrinol..

[117] Singh P., Paramanik V. (2022). Neuromodulating roles of estrogen and phytoestrogens in cognitive therapeutics through epi-genetic modifications during aging. Front. Aging Neurosci..

[118] Wilson M.E., Sengoku T. (2013). Developmental regulation of neuronal genes by DNA methylation: Environment influences. Int. J. Dev. Neurosci..

[119] Lavebratt C., Almgren M., Ekström T.J. (2011). Epigenetic regulation in obesity. Int. J. Obes..

[120] Bove R.M., Patrick E., Aubin C.M., Srivastava G., Schneider J.A., Bennett D.A., De Jager P.L., Chibnik L.B. (2018). Reproductive period and epigenetic modifications of the oxidative phosphorylation pathway in the human prefrontal cortex. PLoS ONE.

[121] Toro C.A., Aylwin C.F., Lomniczi A. (2018). Hypothalamic epigenetics driving female puberty. J. Neuroendocr..

[122] Lomniczi A., Loche A., Castellano J.M., Ronnekleiv O.K., Bosch M., Kaidar G., Knoll J.G., Wright H., Pfeifer G.P., Ojeda S.R. (2013). Epigenetic control of female puberty. Nat. Neurosci..

[123] Lomniczi A., Wright H., Ojeda S.R. (2015). Epigenetic regulation of female puberty. Front. Neuroendocr..

[124] Wu Y., Peterson K.E., Sánchez B.N., Dolinoy D.C., Mercado-Garcia A., Téllez-Rojo M.M., Goodrich J.M. (2018). Association of blood leukocyte DNA methylation at LINE-1 and growth-related candidate genes with pubertal onset and progression. Epigenetics.

[125] Ivanova E., Le Guillou S., Hue-Beauvais C., Le Provost F. (2021). Epigenetics: New Insights into Mammary Gland Biology. Genes.

[126] Stueve T.R., Wolff M.S., Pajak A., Teitelbaum S.L., Chen J. (2014). CYP19A1 promoter methylation in saliva associated with milestones of pubertal timing in urban girls. BMC Pediatr..

[127] Leka-Emiri S., Chrousos G.P., Kanaka-Gantenbein C. (2017). The mystery of puberty initiation: Genetics and epigenetics of idiopathic central precocious puberty (ICPP). J. Endocrinol. Investig..

[128] Shim Y.S., Lee H.S., Hwang J.S. (2022). Genetic factors in precocious puberty. Clin. Exp. Pediatr..

[129] Jones P.R., Voisin S., Nolan B.J., Landen S., Jacques M., Newell B., Zwickl S., Cook T., Wong A., Ginger A. (2022). Uncovering the effects of gender affirming hormone therapy on skeletal muscle and epigenetics: Protocol for a prospective matched cohort study in transgender individuals (the GAME study). BMJ Open.

[130] Gao F., Das S.K. (2014). Epigenetic regulations through DNA methylation and hydroxymethylation: Clues for early pregnancy in decidualization. Biomol. Concepts.

[131] A Michalczyk A., Janus E.D., Judge A., Ebeling P.R., Best J.D., Ackland M.J., Asproloupos D., A Dunbar J. (2018). Transient epigenomic changes during pregnancy and early postpartum in women with and without type 2 diabetes. Epigenomics.

[132] Andrawus M., Sharvit L., Atzmon G. (2022). Epigenetics and Pregnancy: Conditional Snapshot or Rolling Event. Int. J. Mol. Sci..

[133] Zuccarello D., Sorrentino U., Brasson V., Marin L., Piccolo C., Capalbo A., Andrisani A., Cassina M. (2022). Epigenetics of pregnancy: Looking beyond the DNA code. J. Assist. Reprod. Genet..

[134] Das J., Maitra A. (2021). Maternal DNA Methylation During Pregnancy: A Review. Reprod. Sci..

[135] Munro S.K., Balakrishnan B., Lissaman A.C., Gujral P., Ponnampalam A.P. (2021). Cytokines and pregnancy: Potential regulation by histone deacetylases. Mol. Reprod. Dev..

[136] Huang E., Chen L. (2023). RNA N6-methyladenosine modification in female reproductive biology and pathophysiology. Cell Commun. Signal..

[137] Ning J., Yan J., Yang H. (2023). Exploring the role of m6A modification in the great obstetrical syndromes. J. Matern. Neonatal Med..

[138] Rijnkels M., Freeman-Zadrowski C., Hernandez J., Potluri V., Wang L., Li W., Lemay D.G. (2013). Epigenetic Modifications Unlock the Milk Protein Gene Loci during Mouse Mammary Gland Development and Differentiation. PLoS ONE.

[139] Huh S.J., Clement K., Jee D., Merlini A., Choudhury S., Maruyama R., Yoo R., Chytil A., Boyle P., Ran F.A. (2015). Age- and Pregnancy-Associated DNA Methylation Changes in Mammary Epithelial Cells. Stem Cell Rep..

[140] Xue Q., Huang Y., Cheng C., Wang Y., Liao F., Duan Q., Wang X., Miao C. (2023). Progress in epigenetic regulation of milk synthesis, with particular emphasis on mRNA regulation and DNA methylation. Cell Cycle.

[141] VanHouten J.N., Wysolmerski J.J. (2003). Low Estrogen and High Parathyroid Hormone-Related Peptide Levels Contribute to Accelerated Bone Resorption and Bone Loss in Lactating Mice. Endocrinology.

[142] Torres D.A., Freitas M.B., Gonçalves R.V. (2018). Changes in bone turnover and calcium homeostasis during pregnancy and lactation in mammals: A meta-analysis. Reprod. Fertil. Dev..

[143] Carsote M., Turturea M.R., Valea A., Buescu C., Nistor C., Turturea I.F. (2023). Bridging the Gap: Pregnancy—And Lactation—Associated Osteoporosis. Diagnostics.

[144] Lee Y., Hanevik H.I. (2022). Early menopause and epigenetic biomarkers of ageing. Reprod. Biomed. Online.

[145] Jintaridth P., Tungtrongchitr R., Preutthipan S., Mutirangura A. (2013). Hypomethylation of Alu Elements in Post-Menopausal Women with Osteoporosis. PLoS ONE.

[146] Ulrich C.M., Toriola A.T., Koepl L.M., Sandifer T., Poole E.M., Duggan C., McTiernan A., Issa J.-P.J. (2012). Metabolic, hormonal and immunological associations with global DNA methylation among postmenopausal women. Epigenetics.

[147] Zhong X., Song Z., Gao P., Li M., Ning Z., Song X. (2022). Identification of an Epigenetic Signature for Coronary Heart Disease in Postmenopausal Women’s PBMC DNA. Mediat. Inflamm..

[148] Calaf Alsina J.C. (1997). Benefits of hormone replacement therapy—Overview and update. Int. J. Fertil. Women’s Med..

[149] Kreatsoulas C., Anand S.S. (2013). Menopausal hormone therapy for the primary prevention of chronic conditions. U.S. Preventive Services Task Force recommendation statement. Pol. Arch. Intern. Med..

[150] Lobo R.A., Pickar J.H., Stevenson J.C., Mack W.J., Hodis H.N. (2016). Back to the future: Hormone replacement therapy as part of a prevention strategy for women at the onset of menopause. Atherosclerosis.

[151] Cagnacci A., Venier M. (2019). The Controversial History of Hormone Replacement Therapy. Medicina.

[152] Beral V., Banks E., Reeves G., Appleby P. (1999). Use of HRT and the subsequent risk of cancer. J. Epidemiol. Biostat..

[153] Tavani A., La Vecchia C. (1999). The Adverse Effects of Hormone Replacement Therapy. Drugs Aging.

[154] Carson D.S. (1996). Menopause and Osteoporosis: The Role of HRT. J. Am. Pharm. Assoc..

[155] McClung M.R. (2003). Prevention and management of osteoporosis. Best Pr. Res. Clin. Endocrinol. Metab..

[156] Salpeter S.R., Walsh J.M.E., Ormiston T.M., Greyber E., Buckley N.S., Salpeter E.E. (2005). Meta-analysis: Effect of hormone-replacement therapy on components of the metabolic syndrome in postmenopausal women. Diabetes Obes. Metab..

[157] Stevenson T.E.J., Brincat M.P., Pollacco J., Stevenson J.C. (2023). Effect of hormone replacement therapy on intervertebral disc height. Climacteric.

[158] Sourouni M., Sourouni M., Kiesel L., Kiesel L. (2023). Menopausal Hormone Therapy and the Breast: A Review of Clinical Studies. Breast Care.

[159] de Klerk B.M., Schiphof D., Groeneveld F.P.M.J., Koes B.W., van Osch G.J.V.M., van Meurs J.B.J., Bierma-Zeinstra S.M.A. (2008). Limited evidence for a protective effect of unopposed oestrogen therapy for osteoarthritis of the hip: A systematic review. Rheumatology.

[160] Bendale D.S., Karpe P.A., Chhabra R., Shete S.P., Shah H., Tikoo K. (2013). 17-β Oestradiol prevents cardiovascular dysfunction in post-menopausal metabolic syndrome by affecting SIRT1/AMPK/H3 acetylation. Br. J. Pharmacol..

[161] Hashemzadeh M., Romo R., Arreguin J.M., Movahed M.R. (2021). The effects of estrogen and hormone replacement therapy on cardiovascular systems. Futur. Cardiol..

[162] Mills Z.B., Faull R.L.M., Kwakowsky A. (2023). Is Hormone Replacement Therapy a Risk Factor or a Therapeutic Option for Alzheimer’s Disease?. Int. J. Mol. Sci..

[163] Bahl A., Pöllänen E., Ismail K., Sipilä S., Mikkola T.M., Berglund E., Lindqvist C.M., Syvänen A.-C., Rantanen T., Kaprio J. (2015). Hormone Replacement Therapy Associated White Blood Cell DNA Methylation and Gene Expression are Associated with within-Pair Differences of Body Adiposity and Bone Mass. Twin Res. Hum. Genet..

[164] Hilser J.R., Hartiala J.A., Sriprasert I., Kono N., Cai Z., Karim R., DeYoung J., Mack W.J., Hodis H.N., Allayee H. (2022). Effect of menopausal hormone therapy on methylation levels in early and late postmenopausal women. Clin. Epigenetics.

[165] Noronha N.Y., Rodrigues G.d.S., Noma I.H.Y., Brandao C.F.C., Rodrigues K.P., Bruno A.C., Sae-Lee C., Watanabe L.M., Pinhel M.A.d.S., Schineider I.M. (2022). 14-weeks combined exercise epigenetically modulated 118 genes of menopausal women with prediabetes. Front. Endocrinol..

[166] Rodrigues G.d.S., Noronha N.Y., Noma I.H.Y., de Lima J.G.R., Sobrinho A.C.d.S., Pinhel M.A.d.S., de Almeida M.L., Watanabe L.M., Nonino C.B., Júnior C.R.B. (2024). 14-Week exercise training modifies the DNA methylation levels at gene sites in non-Alzheimer’s disease women aged 50 to 70 years. Exp. Gerontol..

[167] Rodriguez C.P., Ogunmoroti O., Minhas A.S., Vaidya D., Kazzi B., Osibogun O., Whelton S., Kovell L.C., Harrington C.M., Honigberg M.C. (2023). Female-specific risk factors of parity and menopause age and risk of carotid plaque: The multi-ethnic study of atherosclerosis. Am. J. Cardiovasc. Dis..

[168] Lai P.M.R., Jimenez M., Du R., Rexrode K. (2022). Association of Reproductive Life Span and Age at Menopause with the Risk of Aneurysmal Subarachnoid Hemorrhage. Neurology.

[169] Hwang S., Kang S.W., Choi K.J., Son K.Y., Lim D.H., Shin D.W., Choi D., Kim S.J. (2022). Early menopause is associated with increased risk of retinal vascular occlusions: A nationwide cohort study. Sci. Rep..

[170] Han S.-L., Liu D.-C., Tan C.-C., Tan L., Xu W. (2023). Male- and female-specific reproductive risk factors across the lifespan for dementia or cognitive decline: A systematic review and meta-analysis. BMC Med..

[171] Xie Q., Xu H., Wan Q. (2021). Correlation between parity and metabolic syndrome in Chinese women aged 40 years and older: The Reaction study. BMC Endocr. Disord..

[172] Hussain S.M., Cicuttini F.M., Alyousef B., Wang Y. (2018). Female hormonal factors and osteoarthritis of the knee, hip and hand: A narrative review. Climacteric.

[173] Liu B., Balkwill A., Cooper C., Roddam A., Brown A., Beral V., on behalf of the Million Women Study Collaborators (2008). Reproductive history, hormonal factors and the incidence of hip and knee replacement for osteoarthritis in middle-aged women. Ann. Rheum. Dis..

[174] Minami T., Muramatsu M., Kume T. (2019). Organ/Tissue-Specific Vascular Endothelial Cell Heterogeneity in Health and Disease. Biol. Pharm. Bull..

[175] Parab S., Setten E., Astanina E., Bussolino F., Doronzo G. (2023). The tissue-specific transcriptional landscape underlines the involvement of endothelial cells in health and disease. Pharmacol. Ther..

[176] Peretz A., Loyfer N., Piyanzin S., Ochana B.L., Neiman D., Magenheim J., Klochendler A., Drawshy Z., Fox-Fisher I., Fridlich O. (2023). The DNA methylome of human vascular endothelium and its use in liquid biopsies. Med.

[177] Palikuqi B., Nguyen D.-H.T., Li G., Schreiner R., Pellegata A.F., Liu Y., Redmond D., Geng F., Lin Y., Gómez-Salinero J.M. (2020). Adaptable haemodynamic endothelial cells for organogenesis and tumorigenesis. Nature.

[178] Shen Z., Bei Y., Lin H., Wei T., Dai Y., Hu Y., Zhang C., Dai H. (2023). The role of class IIa histone deacetylases in regulating endothelial function. Front. Physiol..

[179] McDougall J.J., Giles R.W., Bray R.C., Hart D.A. (1998). Pregnancy-induced changes in rabbit medial collateral ligament vas-oregulation. Am. J. Physiol..

[180] McDougall J.J., Bray R.C., Hart D.A. (2000). Late gestational changes in sympathomimetic sensitivity in primigravid rabbit ligaments. Can. J. Physiol Pharmacol..

[181] Chelly A., Bouzid A., Neifar F., Kammoun I., Tekari A., Masmoudi S., Chtourou H., Rebai A. (2023). Effect of Aerobic/Strength Training on RANKL Gene DNA Methylation Levels. J. Phys. Act. Health.

[182] Proia P., Rossi C., Alioto A., Amato A., Polizzotto C., Pagliaro A., Kuliś S., Baldassano S. (2023). MiRNAs Expression Modulates Osteogenesis in Response to Exercise and Nutrition. Genes.

[183] Chen X., Zhu X., Wei A., Chen F., Gao Q., Lu K., Jiang Q., Cao W. (2021). Nrf2 epigenetic derepression induced by running exercise protects against osteoporosis. Bone Res..

[184] Elsner V.R., Fraga I., Weber C., Galiano W.B., Iraci L., Wohlgemuth M., Morales G., Cercato C., Rodriguez J., Pochmann D. (2021). Effects of a multimodal exercise protocol on functional outcomes, epigenetic modulation and brain-derived neurotrophic factor levels in institutionalized older adults: A quasi-experimental pilot study. Neural Regen. Res..

[185] Zimmer P., Bloch W. (2015). Physical exercise and epigenetic adaptations of the cardiovascular system. Herz.

[186] Zhang X., Gao F. (2021). Exercise improves vascular health: Role of mitochondria. Free Radic. Biol. Med..

[187] Mitsiou G., Tokmakidis S.P., Dinas P.C., Smilios I., Nanas S. (2022). Endothelial progenitor cell mobilization based on exercise volume in patients with cardiovascular disease and healthy individuals: A systematic review and meta-analysis. Eur. Hear. J. Open.

[188] Yang Q., Chen S., Wang X., Yang X., Chen L., Huang T., Zheng Y., Zheng X., Wu X., Sun Y. (2023). Exercise Mitigates Endothelial Pyroptosis and Atherosclerosis by Downregulating NEAT1 Through N6-Methyladenosine Modifications. Arter. Thromb. Vasc. Biol..

[189] Espin-Garcia O., Baghel M., Brar N., Whittaker J.L., Ali S.A. (2022). Can genetics guide exercise prescriptions in osteoarthritis?. Front. Rehabil. Sci..

[190] Barros L., Eichwald T., Solano A.F., Scheffer D.d.L., da Silva R.A., Gaspar J.M., Latini A. (2019). Epigenetic modifications induced by exercise: Drug-free intervention to improve cognitive deficits associated with obesity. Physiol. Behav..

[191] Barha C.K., Falck R.S., Skou S.T., Liu-Ambrose T. (2020). Personalising exercise recommendations for healthy cognition and mobility in aging: Time to address sex and gender (Part 1). Br. J. Sports Med..

[192] Barha C.K., Falck R.S., Skou S.T., Liu-Ambrose T. (2020). Personalizing exercise recommendations for healthy cognition and mobility in aging: Time to consider ones’ pre-existing function and genotype (Part 2). Br. J. Sports Med..

[193] Balbim G.M., Falck R.S., Barha C.K., Starkey S.Y., Bullock A., Davis J.C., Liu-Ambrose T. (2022). Effects of exercise training on the cognitive function of older adults with different types of dementia: A systematic review and meta-analysis. Br. J. Sports Med..

[194] Rossman M.J., Kaplon R.E., Hill S.D., McNamara M.N., Santos-Parker J.R., Pierce G.L., Seals U.R., Donato A.J. (2017). Endothelial cell senescence with aging in healthy humans: Prevention by habitual exercise and relation to vascular endothelial function. Am. J. Physiol. Circ. Physiol..

[195] Sujkowski A.L., Hong L., Wessells R., Todi S.V. (2021). The protective role of exercise against age-related neurodegeneration. Ageing Res. Rev..

[196] Königstein K., Dipla K., Zafeiridis A. (2023). Training the Vessels: Molecular and Clinical Effects of Exercise on Vascular Health—A Narrative Review. Cells.

[197] Meng J., Geng Q., Jin S., Teng X., Xiao L., Wu Y., Tian D. (2023). Exercise protects vascular function by countering senescent cells in older adults. Front. Physiol..

[198] Spartano N.L., Wang R., Yang Q., Chernofsky A., Murabito J.M., Levy D., Vasan R.S., DeCarli C., Maillard P., Seshadri S. (2023). Association of Physical Inactivity with MRI Markers of Brain Aging: Assessing Mediation by Cardiometabolic and Epigenetic Factors. J. Alzheimer’s Dis..

[199] Ma C.-L., Ma X.-T., Wang J.-J., Liu H., Chen Y.-F., Yang Y. (2017). Physical exercise induces hippocampal neurogenesis and prevents cognitive decline. Behav. Brain Res..

[200] Kim-Ha J., Kim Y.-J. (2016). Age-related epigenetic regulation in the brain and its role in neuronal diseases. BMB Rep..

[201] Recchioni R., Marcheselli F., Antonicelli R., Lazzarini R., Mensà E., Testa R., Procopio A.D., Olivieri F. (2016). Physical activity and progenitor cell-mediated endothelial repair in chronic heart failure: Is there a role for epigenetics?. Mech. Ageing Dev..

[202] Georgieva I., Tchekalarova J., Iliev D., Tzoneva R. (2023). Endothelial Senescence and Its Impact on Angiogenesis in Alzheimer’s Disease. Int. J. Mol. Sci..

[203] Barha C.K., Starkey S.Y., Hsiung G.Y.R., Tam R., Liu-Ambrose T. (2023). Aerobic exercise improves executive functions in females, but not males, without the BDNF Val66Met polymorphism. Biol. Sex Differ..

[204] Barha C.K., Best J.R., Rosano C., Yaffe K., Catov J.M., Liu-Ambrose T. (2022). Walking for Cognitive Health: Previous Parity Moderates the Relationship between Self-Reported Walking and Cognition. J. Gerontol. Ser. A.

[205] Rode B., Shi J., Endesh N., Drinkhill M.J., Webster P.J., Lotteau S.J., Bailey M.A., Yuldasheva N.Y., Ludlow M.J., Cubbon R.M. (2017). Piezo1 channels sense whole body physical activity to reset cardiovascular homeostasis and enhance performance. Nat. Commun..

[206] Bartoli F., Debant M., Chuntharpursat-Bon E., Evans E.L., Musialowski K.E., Parsonage G., Morley L.C., Futers T.S., Sukumar P., Bowen T.S. (2022). Endothelial Piezo1 sustains muscle capillary density and contributes to physical activity. J. Clin. Investig..

[207] Zong B., Yu F., Zhang X., Pang Y., Zhao W., Sun P., Li L., Zong B., Yu F., Zhang X. (2023). Mechanosensitive Piezo1 channel in physiology and pathophysiology of the central nervous system. Ageing Res. Rev..

[208] Zi H., Peng X., Cao J., Xie T., Liu T., Li H., Bu J., Du J., Li J. (2024). Piezo1-dependent regulation of pericyte proliferation by blood flow during brain vascular development. Cell Rep..

[209] Davies P.F., Manduchi E., Jiménez J.M., Jiang Y.-Z. (2017). Biofluids, cell mechanics and epigenetics: Flow-induced epigenetic mechanisms of endothelial gene expression. J. Biomech..

[210] Sharma G., Sultana A., Abdullah K.M., Pothuraju R., Nasser M.W., Batra S.K., Siddiqui J.A. (2024). Epigenetic regulation of bone remodeling and bone metastasis. Semin. Cell Dev. Biol..

[211] Castro-Muñoz L.J., Ulloa E.V., Sahlgren C., Lizano M., De La Cruz-Hernández E., Contreras-Paredes A. (2023). Modulating epigenetic modifications for cancer therapy (Review). Oncol. Rep..

[212] Kim A., Mo K., Kwon H., Choe S., Park M., Kwak W., Yoon H. (2023). Epigenetic Regulation in Breast Cancer: Insights on Epidrugs. Epigenomes.

[213] Martínez-Iglesias O., Naidoo V., Carrera I., Corzo L., Cacabelos R. (2023). Natural Bioactive Products as Epigenetic Modulators for Treating Neurodegenerative Disorders. Pharmaceuticals.

[214] Gladkova M.G., Leidmaa E., Anderzhanova E.A. (2023). Epidrugs in the Therapy of Central Nervous System Disorders: A Way to Drive on?. Cells.

[215] Gladwell L.R., Ahiarah C., Rasheed S., Rahman S.M., Choudhury M. (2023). Traditional therapeutics and potential epidrugs for CVD: Why not both?. Life.

[216] Musolino E., Pagiatakis C., Serio S., Borgese M., Gamberoni F., Gornati R., Bernardini G., Papait R. (2022). The Yin and Yang of epigenetics in the field of nanoparticles. Nanoscale Adv..

[217] Sharma S., Bhonde R. (2023). Epigenetic Modifiers as Game Changers for Healthy Aging. Rejuvenation Res..

[218] Farani M.R., Sarlak M., Gholami A., Azaraian M., Binabaj M.M., Kakavandi S., Tambuwala M.M., Taheriazam A., Hashemi M., Ghasemi S. (2023). Epigenetic drugs as new emerging therapeutics: What is the scale’s orientation of application and challenges?. Pathol.-Res. Pr..

[219] Hart D.A., Zernicke R.F. (2020). Optimal Human Functioning Requires Exercise across the Lifespan: Mobility in a 1g Environment Is Intrinsic to the Integrity of Multiple Biological Systems. Front. Physiol..

[220] Ma L., Liu G., Ding M., Zong G., Hu F.B., Willett W.C., Rimm E.B., Manson J.E., Sun Q. (2020). Isoflavone intake and the risk of coronary heart disease in US men and women. Circulation.

[221] Sanchez-Martinez L., Periago M.-J., Garcia-Alonso J., Garcia-Conesa M.-T., Gonzalez-Barrio R. (2021). A systematic review of the cardiometabolic benefits of plant products containing mixed phenolics and polyphenols in postmenopausal women: Insufficient evidence for recommendations to this specific population. Nutrients.

[222] Marini H.R. (2022). Mediterranean diet and soy isoflavones for integrate3d management of menopausal metabolic syndrome. Nutrients.

[223] Calderaro A., Patane G.T., Tellone E., Barreca D., Ficarra S., Misiti F., Lagana G. (2022). The neuroprotective potentiality of flavonoids on Alzheimer’s disease. Int. J. Mol. Sci..

[224] Peng X., Zhu Y., Wu Y., Xiang X., Deng M., Liu L., Li T., Yang G. (2023). Genistein, a soybean isoflavone, promotes wound healing by enhancing endothelial progenitor cell mobilization in rats with hemorrhagic shock. Adv. Biol..

[225] Ganai A.A., Farooqi H. (2015). Bioactivity of genistein: A review of in vitro and in vivo studies. Biomed. Pharmacother..

[226] Yamagata K. (2019). Soy isoflavones inhibit endothelial cell dysfunction and prevent cardiovascular disease. J. Cardiovasc. Pharmacol..

[227] Duan H., Zhang Q., Liu J., Li R., Wang D., Peng W., Wu C. (2021). Suppression of apoptosis in vascular endothelial cell, the promising way for natural medicines to treat atherosclerosis. Pharmacol. Res..

[228] Zhang H., Pang X., Yu H., Zhou H. (2022). Genistein suppresses ox-LDL-elicited oxidative stress and senescence in HUVEs through the SIRT1-p66shc-Foxo3a pathways. J. Biochem. Mol. Toxicol..

[229] Xu K., Qin Q., Yao L., Du X., Zhou K., Wi X., Wang W., Liu C. (2024). Anti-oxidation effect of genistein in vascular endothelial cell after H_2_O_2_ stress. Mol. Med. Rep..

[230] Squadrito F., Marini H., Bitto A., Altavilla D., Polito F., Adamo E.B., D’Anna R., Arcoraci V., Burnett B.P., Minutoli L. (2013). Genistein in the metabolic syndrome: Results of a randomized clinical trial. J. Clin. Endocrinol. Metab..

[231] Saad B., Ghareeb B., Kmail A. (2021). Metabolic and epigenetics action mechanisms of antiobesity medicinal plants and phytochemicals. Evid. Based Complement. Alternat. Med..

[232] Huminiecki L. (2022). Evidence for multilevel chemopreventive activities of natural phenols from functional genomics studies of curcumin, resveratrol, genistein, quercetin, and luteolin. Int. J. Mol. Sci..

[233] Kocabas S., Sanlier N. (2023). A comprehensive overview of the complex relationship between epigenetics, bioactive components, cancer, and aging. Crit. Rev. Food Sci. Nutr..

[234] Acar Y., Agagunduz D., De Cicco P., Capasso R. (2023). Flavonoids: Their putative neurologic roles, epigenetic changes, and gut microbiota alterations in Parkinson’s disease. Biomed. Pharmacother..

[235] Chen P., Wang Y., Chen F., Zhau B. (2024). Epigenetics in obesity: Mechanisms and advances in therapies based on natural products. Pharmacol. Res. Perspect..

